# Long-Term Stability of Ferroelectret Energy Harvesters

**DOI:** 10.3390/ma13010042

**Published:** 2019-12-20

**Authors:** Muhammed Kayaharman, Taylan Das, Gregory Seviora, Resul Saritas, Eihab Abdel-Rahman, Mustafa Yavuz

**Affiliations:** 1 Electrical and Computer Engineering Department, University of Waterloo, 200 University Avenue West, Waterloo, ON N2L 3G1, Canada; 2 Mechanical and Mechatronics Engineering Department, University of Waterloo, 200 University Avenue West, Waterloo, ON N2L 3G1, Canada; taylandas@gmail.com (T.D.); gseviora@uwaterloo.ca (G.S.); myavuz@uwaterloo.ca (M.Y.); 3 Mechanical Engineering Department, Kirikkale University, 71450 Kirikkale, Turkey; 4 Systems Design Engineering Department, University of Waterloo, 200 University Avenue West, Waterloo, ON N2L 3G1, Canada; resulsaritas@gmail.com (R.S.); eihab@uwaterloo.ca (E.A.-R.)

**Keywords:** energy harvesters, ferroelectret, piezoelectric

## Abstract

Cellular polypropylene (PP) has been recently used in energy harvesting applications. In this work, we investigate its viability and long-term stability under various operating conditions. Specifically, the effect of constant stress and stress cycling on output power and long-term stability of ferroelectret energy harvesters is analyzed. Our findings show that after 112 days constant stress significantly increases the piezoelectric charge constant $d_{33}$ and output power from 0.51 μW for a stress-free harvester to 2.71 μW. It also increases the harvester center frequency from 450 to 700 Hz and decreases its optimal resistance from 7 to 5.5 MΩ.

## 1. Introduction

Energy harvesting applications have seen significant interest over recent decades [1] as they have become a viable power source for mobile electronics. Current batteries used for wireless sensors have limited lifetimes. Expensive and time-consuming battery recharging or replacement procedures are required for each depleted battery in a sensor network. They are not even an option for applications in dangerous or inaccessible environments.

Energy harvesters are an appealing option for self-powered and sustainable electronics. Different transduction mechanisms have been used to convert ambient mechanical vibrations into electrical energy [2,3]. Among those mechanisms, piezoelectric energy harvesting has received the most attention [1].

The piezoelectric charge constant $d_{ij}$ and the harvester stiffness *k* are two of the most important attributes that effect their efficiency. High piezoelectric constant and low stiffness are required to increase the efficiency of power transduction. Both of these factors depend on the material properties of piezoelectric material. Current piezoelectric materials with high charge constant, such as lead zirconate titanate (PZT), are also stiff, which limits the harvester displacement [4,5]. More flexible piezoelectrics, such as polyvinylidene fluoride (PVDF), have lower piezoelectric charge constants. PZT is also brittle which makes it liable to degradation in harsh environments.

Recently developed ferroelectrets, such as cellular polypropylene (PP) [5,6] and laminated fluoropolymers, are attractive alternatives to piezoelectrics. They combine a high piezoelectric charge constant with material flexibility, making them better option for energy harvesting applications.

While ferroelectrets display a similar response under stress to piezoelectrics, the underlying reasons are different. Polarization in ferroelectrets results from deformation of charged voids. Polarization in piezoelectrics results from ion displacement [7].

Various ferroelectrets, including PP, polyethyelene terephthalate (PET) and cyclic-olefin copolymer (COC), have been used as sensors [8,9] and loudspeakers [10]. However, PP is the most widely used ferroelectret due to a higher charge constant [11] than all other ferroelectrets.

Mellinger et al. [12] studied the thermal and temporal stability of PP films under elevated temperature and static pressure conditions. They found that at room temperature, the piezoelectric charge constant $d_{33}$ of PP drops significantly in the first ten days before stabilizing to a long-term value. They also found that increasing static pressure reduces the film thickness, increases the elastic stiffness, and decreases the piezoelectric charge constant $d_{33}$ of PP. Wan et al. [13] reported that the piezoelectric charge constant $d_{33}$ of PP increases with time under constant stress, whereas Qu et al. [14] predicted that it would also increase with strain, particularly for strains in excess of 1%. Anton et al. [5] found that the stiffness and strength of thin PP sheets were significantly different in the two planar directions, possibly due to asymmetries in biaxial stretching during manufacturing. Ray et al. [15] found that the piezoelectric charge constant $d_{33}$ drops gradually with the excitation frequency up to 1–10 kHz. Comparative analysis of the PP energy harvesters are depicted in Table 1.

Anton et al. [5] examined the performance of single-layer PP energy harvesters under pre-tension supplied by a shaker. Pondrom et al. [6] compared single-layer and multi-layer PP harvesters under a more realistic base excitation. The output power, in all three cases, was limited to a few tens of micro-Watts. Sessler et al. [16] were able to generate 80 μW output power with a larger-area radiation better performing (higher $d_{33}$) cross-linked multi-layer PP energy harvester. On the other hand, Ray et al. [15] generated 0.45 μW with a 20-layer PP harvester because of a lower $d_{33}$ film. Luo et al. [17,18] harvested energy from the impact of body weight during the heel strike stage of walking sing multi-layer PP harvesters.

Pondrom et al. [19] proposed and validated a model for PP energy harvesters under harmonic base exctiation. Ray et al. [15] independently validated this model. Luo et al. [18] proposed a model for PP energy harvesters under impulse excitation.

Since cellular PP is a voided charged polymer, the charge voids may deform with time under stress. In fact, an important challenge to wide adoption of cellular PP in energy harvesting has been a lack of information about the long-term stability and durability of the material and the performances of energy harvesters made using it. In this work, we fabricated three identical one-layer PP energy harvesters, characterized them, and evaluated their performances over a three-month period under continuous static stress, dynamic stress, and no-stress conditions to study their long-term stability. We also examined whether the long-term response of ferroelectric harvesters followed Pondrom et al.’s [19] model.

## 2. Methods

### 2.1. Mathematical Model

We hypothesize that the response of ferroelectric harvesters will continue to follow that of piezoelectric harvesters over the long-term, as it has been shown to do over the short-term by Pondrom et al. [6,19] and Ray et al. [15]. To test this hypothesis, we compare their mathematical model to our experimental results over the test period here.

**Figure 1:**
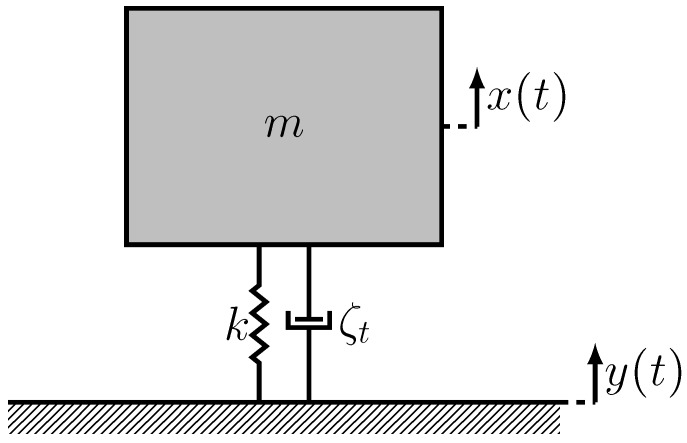
Lumped parameter base excitation model for ferroelectret energy harvester.

Figure 1 presents a lumped-parameter model of the ferroelectret harvester. The motions of the parent body (base excitations) supplying kinetic energy to the harvester are denoted as y(t). The absolute displacement of the seismic mass *m* is denoted as x(t). The relative displacement of the seismic mass with respect to the base is denoted as z(t)=x(t)−y(t). The equation of motion of the harvester can be written as

(1) $$\ddot{z}+2\zeta_{t}\omega \dot{z}+\omega^{2}z=-\ddot{y}$$

where the overdotes denote derivatives with respect to time.

$$\zeta_{t}=\zeta_{m}+\zeta_{e}$$

is the total damping ratio of the harvester, representing the summation of mechanical energy dissipation $\zeta_{m}$ and electrical energy dissipation and harvesting $\zeta_{e}$; and ω is its natural frequency:

(2) $$\omega =\sqrt{\frac{k}{m_{eq}}}.$$

The natural frequency is a function of the harvester effective mass $m_{eq}$ and effective stiffness *k*.

Ignoring the mass of the ferroelectret harvester, the effective mass can be set equal to the seismic mass $m_{eq}=m$. Further, ignoring the stiffer bonding layers, the stiffness of harvester under compressive load, can be written as:

(3) $$\begin{array}{c} k=\frac{EA}{h} \end{array}$$

where *E* is Young’s modulus of PP. Therefore, the natural frequency of the harvester can be written as

(4) $$\omega =\sqrt{\frac{EA}{h\;m}}.$$

Since the excitations of interest in this work are harmonic, we can write

$$y\left(t\right)=Y_{\circ }e^{j\Omega t}$$

where $Y_{\circ }$ and Ω are the base displacement amplitude and frequency. The steady-state displacement of the seismic mass can then be written as

(5) $$z\left(t\right)=\frac{\Omega^{2}Y_{\circ }e^{j\Omega t}}{\omega^{2}-\Omega^{2}+2\zeta_{t}\omega \Omega }.$$

The charge collected on the electrodes of the harvester can be described using the direct piezoelectric equation as the product of the force applied to the PP film *F* and its piezoelectric charge constant

(6) $$\begin{array}{cc} Q_{f} & =d_{33}F=d_{33}kz\\ & =d_{33}\frac{EA}{h}z. \end{array}$$

Since the relative displacement of the seismic mass z(t) varies with time, the PP film can be modeled as a time-varying current source ${\dot{Q}}_{f}$.

**Figure 2:**
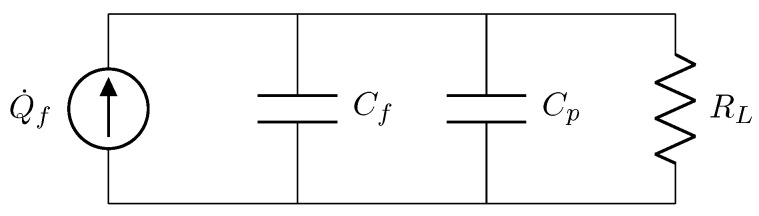
Equivalent circuit for the single-layer PP energy harvester.

Figure 2 shows an equivalent circuit for the PP energy harvester. The PP film is connected in parallel with its internal capacitance

(7) $$C_{f}=\frac{\epsilon A}{h}$$

where ϵ is the electric permittivity of the ferroelectret film. It is also connected in parallel with a parasitic capacitance $C_{p}$ and a resistive load $R_{L}$. Using this equivalent circuit, the harvester output impedance can be calculated as

(8) $$\begin{array}{c} \frac{1}{Z_{eq}}=\frac{1}{R_{L}}+j\Omega \left(C_{f}+C_{p}\right)\\ Z_{eq}=\frac{R_{L}}{1+j\Omega R_{L}\left(C_{f}+C_{p}\right)}. \end{array}$$

### 2.2. Harvester Fabrication

**Figure 3:**
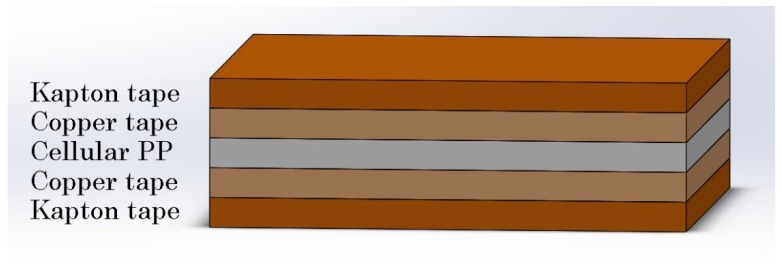
Schematic of the ferroelectret energy harvester.

A non-laminated cellular polypropylene sheet was purchased from EMFIT (Emfitech Ltd, Vaajakoski, Finland). The sheet thickness was h=100±5 μm. The harvester was made of a PP patch with an area of A=1.6×1.6 cm${}^{2}$. Copper tape, 66 μm thick, was attached to top and bottom sides of the PP patch to act as electrodes. The dimensions of the electrodes were smaller than the PP patch to prevent short circuiting. Copper wire connected the electrodes to a load resistor $R_{L}$. Kapton tape, 70 μm thick, was used to electrically isolate the electrodes. A 3D schematic of the one-layer PP energy harvester is shown in Figure 3.

### 2.3. Experimental Procedure

The long-term stability of electrical and electromechanical properties of PP films and PP energy harvesters were observed under three loading conditions: constant-stress, stress-cycling, and stress-free. We fabricated one-layer cellular PP energy harvesters following the fabrication procedure described above. The constant-stress (CS) harvesters were continuously placed under an 8 kg mass throughout the period of the experiment except while being tested on the shaker. The stress-cycled (SC) harvesters were subjected to high amplitude (5 g) base acceleration for 10 min everyday. The stress-free (SF) harvesters were left unloaded when not being tested.

The harvesters were kept under the defined load conditions for 112 days. The open-circuit output voltage of the harvesters was measured every other day for the first 30. After 112 days, the harvesters’ output voltages were measured three times over a period of five days. The capacitance and piezoelectric charge constant for each harvesters were also measured over the experimental course.

#### 2.3.1. Film Characterization

In order to characterize the electromechanical coupling of the PP film, the piezoelectric charge constant $d_{33}$ was measured using a laser interferometry method [20]. It measures the displacement of the ferroelectret film surface *x* under a known voltage signal *V* applied to its electrodes. It then uses the reverse piezoelectric effect to relate applied voltage to measured displacement as

(9) $$d_{33}=\frac{x}{V}.$$

The film displacement was measured with a laser Doppler vibrometer (LDV), Polytec Inc. (Waldbronn, Germany) MSV 400 [21], under sinusoidal voltage signals.

#### 2.3.2. Harvester Characterization

**Figure 4:**
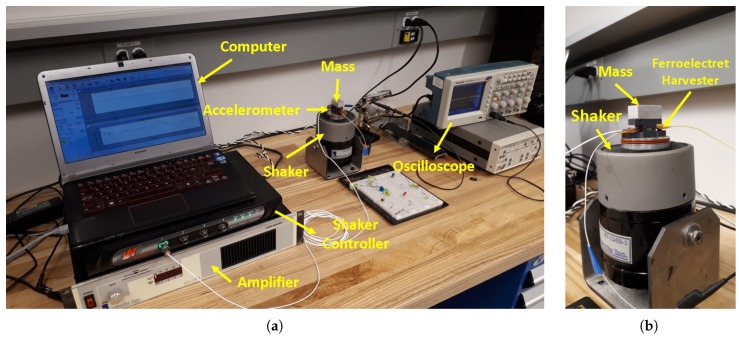
(**a**) The experimental setup for PP energy harvesters. (**b**) A close view of the harvester and shaker.

The experimental setup used to characterize PP harvesters is shown in Figure 4. Vibration Research (Jenison, MI, USA) closed-loop controller (VR9500) was employed to command harmonic base acceleration delivered via Labwork’ s (Costa Mesa, CA, USA) electromagnetic shaker (ET-126-1). An accelerometer was attached to the base to measure its acceleration, thereby closing the control loop. The frequency and amplitude of the base acceleration were set and monitored in the controller’s software interface, Vibration View [22]. Tektronix (Beaverton, OR, USA) oscilloscope (TDC2004C) was used to record and store the voltage measured across the harvester leads (open circuit) and across the load resistance $R_{L}$.

The bottom of the harvester was attached to the shaker base (Figure 4) and a seismic mass was attached to top of the harvester using double-sided tape. To characterize the harvester response, frequency up-sweeps of base acceleration $\ddot{y}$ were carried out in steps of 50 Hz to cover the relevant operation range. After each frequency step, the harvester response was allowed to settle down before an oscilloscope was used to record the open circuit RMS voltage between its leads.

The natural frequency ω of each harvester was determined from its frequency-response curve as the peak voltage frequency. The mechanical damping ratio, representing all non-electrical energy losses, was determined using the half-power bandwidth method as [23]

(10) $$\zeta_{m}=\frac{\omega_{2}-\omega_{1}}{2\omega }$$

where $\omega_{1}$ and $\omega_{2}$ are the frequencies corresponding to $1/\sqrt{2}$ of the peak voltage.

According to impedance matching theorem [24], peak output power for a given excitation level $\Omega^{2}Y_{\circ }$ is realized when the input impedance (mechanical losses)

(11) $$Z_{m}=2\zeta_{m}\sqrt{\frac{k}{m}}$$

and output impedance $Z_{e}$ of the harvester are set equal to each other. This relationship can be expressed as:

(12) $$2\zeta_{m}\sqrt{\frac{k}{m}}=\frac{R_{opt}}{1+j\Omega R_{opt}\left(C_{f}+C_{p}\right)},$$

where $R_{opt}$ is the load resistance that satisfies this condition.

We found the optimal load resistance $R_{opt}$ by varying the magnitude of the resistive load $R_{L}$ under constant base excitation $\ddot{y}$ and measuring the voltage across it using the oscilloscope. The output power was calculated as

(13) $$P=\frac{V_{\mathrm{RMS}}^{2}}{R_{L}}.$$

The optimal resistance $R_{opt}$ was determined as that corresponding to peak power.

## 3. RESULTS

### 3.1. Characterization of Cellular Polypropylene

The piezoelectric charge constants of the CS and SF films were measured after 112 days of energy harvesting. Unbiased harmonic voltage signals with amplitudes of 30, 45, 60, 75, and 90 V and a frequency of Ω=1 kHz were applied to each of the PP films. The amplitude of the film surface velocity was measured in the frequency-domain using the LDV. The displacement amplitude was then calculated as:

(14) $$x=\frac{\dot{x}}{\Omega }.$$

**Figure 5:**
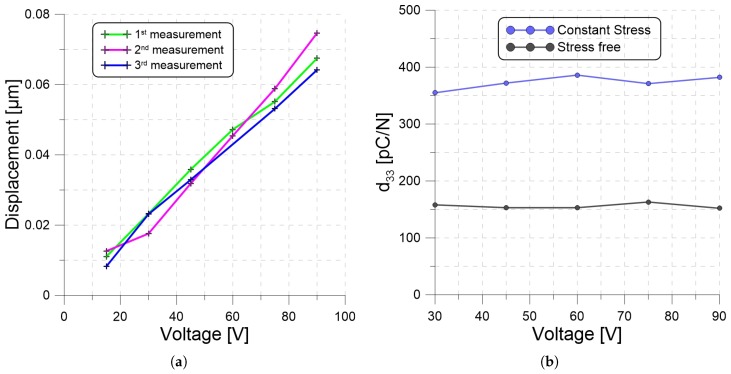
(**a**) Measured displacement amplitude as a function of applied voltage. (**b**) Piezoelectric charge constant $d_{33}$ of the PP films under constant-stress and stress-free loading conditions.

The experiment was repeated three times for each waveform. The results for the CS film are shown in Figure 5a. The average values of displacement were then substituted into Equation (6) to calculate the piezoelectric charge constant $d_{33}$. The results are shown in Figure 5b.

We found that the charge constant was almost insensitive to voltage variation but significantly different for the two loading conditions with $d_{33}=155.8\pm 4.17$ pC/N for the SF film and $d_{33}=370.6\pm 10.76$ pC/N for the CS film. Although no force was applied during testing, the charge constant of the CS film was elevated consistently with the predictions of Qu et al. [14] for films under strain. In their case, they predicted that straining a PP film by 10%, would increase the charge constant from a strain-free value of 200 pC/N to 357 pC/N. We postulate that the long-term strain the CS film experienced results in a plastic deformation that maintains the time-dependent (viscoelastic) enhancement of the charge constant observed by Wan et al. [13].

Our results are also consistent with the predictions of Qu et al. [14] that the charge constant was an anti-symmetric function of DC voltage, increasing and decreasing at the same rate with positive and negative voltage differences, respectively. In our case, the test waveform had a zero-mean; therefore, eliminating the dependence of $d_{33}$ on DC voltage.

### 3.2. Harvester Characterization

We investigated the impact of applying the three loading conditions for 112 days on the performance of PP harvesters by measuring their capacitance and output voltage values over the experiment’s duration.

**Figure 6:**
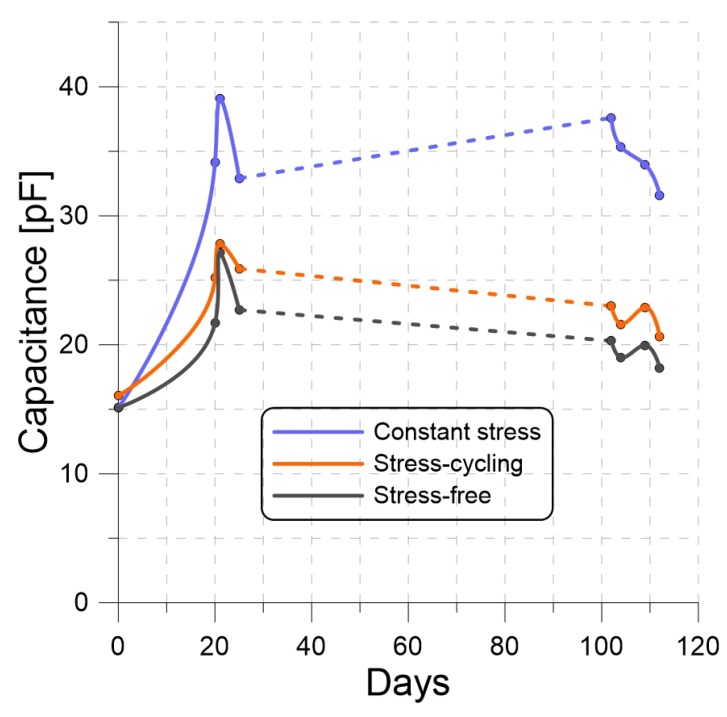
Capacitance of the CS, SC, and SF PP films as functions of time.

We measured the capacitance of each of the CS, SC, and SF PP films, while unloaded, at various points in time over the duration of the experiment. The results are presented as functions of time in Figure 6. The CS film capacitance was consistently higher than that of the SC film, which was in turn higher than that of the SF film. This is consistent with our hypothesis that long-term loading beyond the elastic limit, whether constant or cyclic, builds in to plastically deform PP films.

**Figure 7:**
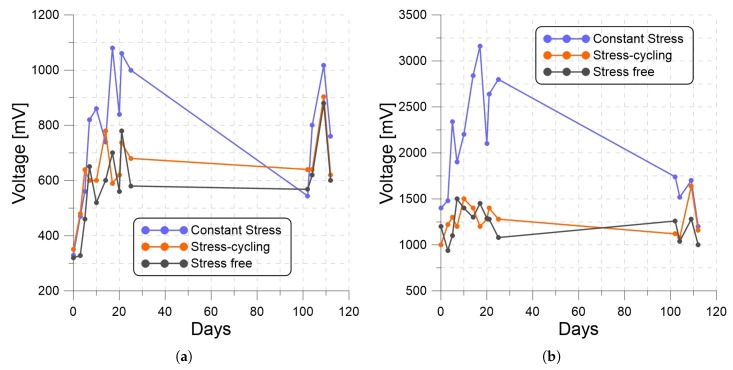
The open-circuit voltage of the 250 g PP harvesters under base acceleration amplitudes of (**a**) 1 g and (**b**) 1.8 g with a frequency of 10 Hz.

We also measured the open-circuit RMS voltages of three harvesters constituted out of a 250 g seismic mass and the CS, SC, and SF PP films at various points in time over the duration of the experiment. The harvesters were excited with two base acceleration amplitudes, 1 g and 1.8 g, at the same frequency, 10 Hz. The results are presented as functions of time in Figure 7. The voltage of the CS harvester was consistently larger than those of the SC and SF harvesters because of its larger piezoelectric charge constant $d_{33}$. Also, increasing the excitation amplitude increased the size of the seismic mass motions z(t), thereby collecting more charge and RMS voltage per excitation period, as indicated by Equation (6).

While the piezoelectric charge constant $d_{33}$ varied due to the loading conditions, it was stable over the experiment duration. On the other hand, the capacitance and, as result, voltage of the harvesters varied in the same period. We postulate that these variations are related to plastic deformation over that period which resulted in changes in the film stiffness.

**Figure 8:**
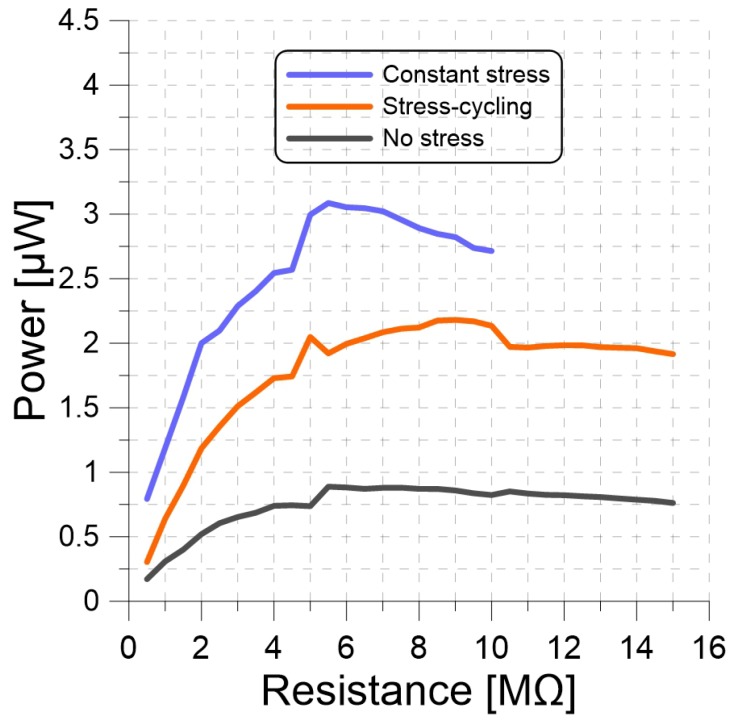
Output power of three PP harvesters constituted out of a 250 g seismic mass and CS, SC, and SF films under base acceleration amplitude and frequency of 1 g and 220 Hz as functions of the load resistance $R_{L}$.

Next, we determined the optimal resistances for three PP harvesters constituted out of a 250 g seismic mass and the CS, SC, and SF films. The harvesters were subjected to a base acceleration amplitude and frequency of 1 g and 220 Hz while the output voltage was measured across resistive loads $R_{L}$ varying in steps of 0.5 MΩ from 0.5 to 15 MΩ. The generated power was calculated from Equation (13) and is shown, for all three harvesters, in Figure 8.

The CS harvester had the highest peak output power at 3.09 μW followed by the SC harvester at 2.18 μW. The SF harvester had the lowest peak output power at 0.87 μW. We note that this does not necessarily represent the maximum attainable power, since we did not attempt to excite the harvesters at resonance. The optimal resistance was found to be $R_{opt}=5.5\;M\Omega$ for the CS harvester, 9 MΩ for the SC harvester, and 7 MΩ for the SF harvester.

Finally, we determined the frequency response for six harvesters, three constituted out of a 25 g seismic mass and CS, SC, and SF films. and three constituted out of a 250 g seismic mass and CS, SC, and SF films. The base acceleration amplitude was set to 1 g for all harvesters. The frequency sweeps of the 25 g harvesters were carried out over the frequency range 20 to 1 kHz and those of the 250 g harvesters were carried out over the frequency range 20 to 500 Hz. The CS and SF harvesters were to a $R_{L}=5.5\;M\Omega$ load resistor, whereas the SC harvesters were connected to a $R_{L}=8.75\;M\Omega$ load resistor.

**Figure 9:**
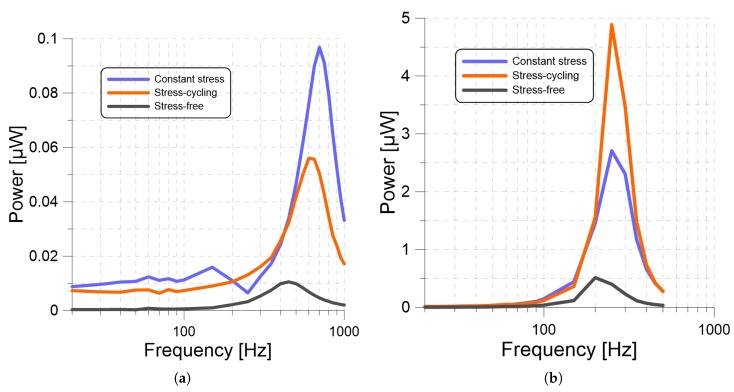
The frequency-response curves of the output power for the (**a**) 25 g and (**b**) 250 g PP harvesters employing CS, SC, and SF films under a base acceleration amplitude of 1 g.

The frequency-response curves of the three 25 g harvesters are shown in Figure 9a. Although all three harvesters had identical masses and areas, their natural frequencies were different. We used Equation (3) to estimate the stiffness ratio of the PP films from the ratio of their natural frequencies as

(15) $$\frac{k_{i}}{k_{j}}=\frac{{\left(E/h\right)}_{i}}{{\left(E/h\right)}_{j}}=\frac{\omega_{i}^{2}}{\omega_{j}^{2}}.$$

The stiffness ratio reduces, in this case, to the ratios of Young’s modulus to thickness. The stiffness ratio of the CS to SC films was found to be 1.36 and the stiffness ratio of the CS to SF films was found to be 2.42. These findings confirm our hypothesis that long-term constant-stress causes plastic deformations that increase the stiffness of PP films. We also found that stress-cycling has a similar, although quantitatively smaller, effect.

The frequency-response curves of the 250 g harvesters, Figure 9b, reflect a downward shift in the natural frequency and more than an order-of-magnitude increase in output power compared to the 25 g harvesters due to a one-order-of-magnitude increase in the seismic mass. We note that the 50 Hz step used to evaluate the frequency response was insufficient to accurately resolve the magnitude or location of the peaks on the frequency-response curves due to the lower natural frequency of the 250 g harvesters.

### 3.3. Model Validation

To validate the ferroelectric harvester model, we used it to predict the output power of two PP harvesters made out of m=25 g and m=250 g, seismic masses, and a SF film under a base acceleration with an amplitude of 1 g, while the frequency was swept over the ranges 10 to 1000 Hz and 10 to 500 Hz, respectively. We also measured experimentally, the output voltages of these harvesters across a $R_{L}=5.5\;M\Omega$ load resistor.

The total damping ratio was estimated from the measured frequency-response curves, as per subsection Equation (10), to be $\zeta_{t}=0.4$ for the 25 g harvester and $\zeta_{t}=0.3$ for the 250 g harvester.

The relative displacement z(t) for each harvester was predicted by inserting these values in the equation of motion, Equation (1), and integrating it over time for a given base excitation frequency *f*. Long-time integration was carried out in using Mathematica until the response settled down to its steady-state value. The generated charge $Q_{f}\left(t\right)$ was calculated from Equation (6) and the RMS of the current passing through the resistor was calculated over an excitation period T=1/f as

$$I_{\mathrm{RMS}}=f\int_{0}^{T}\dot{Q}\left(t\right)dt.$$

The output power of the harvester was calculated as

(16) $$P=I_{\mathrm{RMS}}^{2}R_{L}.$$

**Figure 10:**
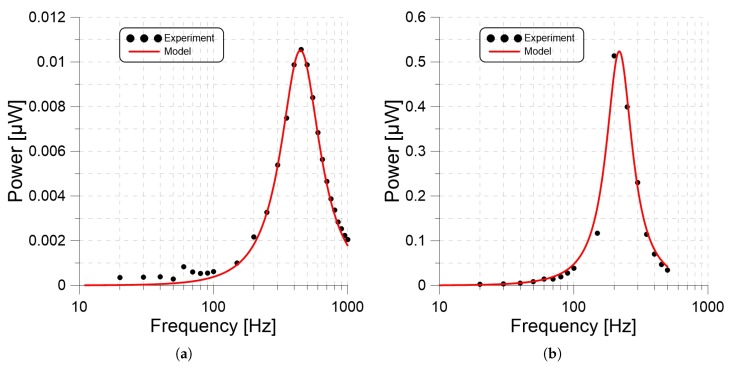
The experimental and analytical frequency-response curves of PP harvesters constituted out of (**a**) 25 g and (**b**) 250 g seismic masses and a SF film under a base acceleration with amplitude of 1 g.

Varying the frequency over the excitation range, the analytical frequency-response curves for the 25 g and 250 g harvesters were obtained. Those curves are compared in Figure 10 (red lines) with the experimental frequency-response curves (black dots). We found close agreement in both cases, confirming the validity of the lumped parameter model.

Comparing the frequency-response curves in Figure 9, we find that the ratios of the natural frequencies of the 25 g to 250 g harvesters are 2.25 for the SF films, 2.6 for the SC films, and 2.8 for the CS films. On the other hand, their ratios as predicted by the lumped-mass model can be written as

(17) $$\frac{\omega_{25}}{\omega_{250}}=3.16\sqrt{\frac{k_{25}}{k_{250}}},$$

which suggests that the stiffness of the heaviest harvester is more than that of the lightest harvester. This could be due to an increase in Young’s modulus, a decrease in film thickness, or both, associated with increased seismic mass. These results show that it is necessary to account for changes in the harvester stiffness due to the seismic mass, since the differences between the ratios predicted from lumped mass model 3.16 and those measured are not negligible.

Further, we note that the discrepancy between lumped-mass model and experimental results is larger for the SF film frequency ratio than it is for the SC film, which in turn is larger than it is for the CS film. This is consistent with our finding above, that the SF film stiffness is lower than that of the SC film, which in turn is less than the stiffness of the CS film. It suggests that the lumped-mass model can be improved by accounting for changes in film stiffness due to the seismic mass as changes in thickness.

## 4. Discussion and Conclusions

In this research, we designed and fabricated three single-layer ferroelectret energy harvesters using cellular PP with a sheet thickness of 100 μm ± 5 μm. Long-term stability of mechanical and electromechanical properties of the harvesters was observed under three different stress loading conditions listed as follows: constant-stress (CS), stress-cycled (SC), and stress-free (SF). Three harvesters were exposed to the aforementioned loading conditions for 112 days. Piezoelectric charge constant $d_{33}$, capacitance *C*, open-circuit output voltage *V*, frequency response, and output power *W* were observed during the course of the experiment. We compared our experimental results with the mathematical model defined for ferroelectret energy harvesters by Pondrom et al. [6,19] and Anton et al. [15].

Our results show the effects of long-term stress loading on mechanical and electrical properties of PP energy harvester. The capacitance *C* value of the CS film was higher than for SC and SF films. And the capacitance *C* value of the SC film was also higher than SF film. These capacitance changes after long-term stress loading might be a result of plastic deformations which also effects the stability of the film. Next, after 112 days of applying three different loading conditions, changes in piezoelectric charge constant $d_{33}$, natural frequency $w_{n}$, and optimum resistance $R_{opt}$ were observed. CS film had a significantly raised charge constant with a value of $d_{33}=370.6\pm 10.76$ pC/N, while the charge constant for the SF film was $d_{33}=155.8\pm 4.17$ pC/N. These results were consistent with the predictions of Qu et al. [14] and the results of Wan et al. [13], which reported an increase in the charge constant due to plastic deformations.

Next, change in optimum resistance was observed by applying a base acceleration and frequency of 1 g and 220 Hz while the output power was calculated for resistive loads $R_{L}$ varying from 0.5 MΩ to 15 MΩ with a 250 g seismic mass. The optimal resistance was 5.5 MΩ for the CS harvester, 9 MΩ for the SC harvester, and 7 MΩ for the SF harvester. In order to analyze the output power as a function of frequency, we measured the frequency response of the harvesters under a base acceleration and a seismic mass of 1 g and 25 g while the output power was calculated for frequencies varying from 20 to 1 kHz. We also measured the output power of the same experiment setup with a seismic mass of 250 g and a frequency range from 20 to 500 Hz. For the 25 g seismic mass, natural frequencies of the CS, SC and SF were 700, 600, and 450 Hz respectively. We used Equation (3) to estimate the stiffness ratio of the PP films from the ratio of natural frequencies $w_{n}$. These changes in the natural frequencies and the stiffness ratio also support our hypothesis that long-term stress causes plastic deformation that increases the stiffness of PP films. We also compared our experimental results with the mathematical model.We found close agreement between the model and experimental frequency-response curves, confirming the validity of the lumped parameter model subject to the modification suggested above. These results suggest that the lumped-mass model is adequate for PP harvesters; however, it can be improved by accounting for changes in film thickness due to the seismic mass.

## Figures and Tables

**Table 1:** Comparative analysis of PP energy harvesters.

Harvester	$d_{33}$ pC/N	Frequency Hz	Area cm${}^{2}$	Layers	Power μW	Seismic Mass gr
Anton [5]	175	60	231.04	1	6	–
Pondrom [6]	250	400	0.25	1	18	40 @ 1.0 g
Sessler [16]	–	400	4	8	80	27 @ 1.0 g
Ray [15]	37.8	90.6	10.08	40	0.89	1000 @ 0.5 g
Luo [17]	295	–	42	80	100	80,000
Luo [18]	295	–	42	1	3.07	80,000
